# Development of Simple Sequence Repeat of *Monochamus alternatus* (Coleoptera: Cerambycidae) Based on Restriction Site-Associated DNA Sequencing

**DOI:** 10.3390/biology13110880

**Published:** 2024-10-29

**Authors:** Jintao Lu, Senzhe Zhang, Jiaxin Liu, Yuhua Zhang, Lijuan Hu, Zhende Yang, Ping Hu

**Affiliations:** 1 Guangxi Colleges and Universities Key Laboratory for Cultivation and Utilization of Subtropical Forest Plantation, Guangxi Key Laboratory of Forest Ecology and Conservation, College of Forestry, Guangxi University, Nanning 530004, China; ljt6212@st.gxu.edu.cn (J.L.); liujiaxin1203@126.com (J.L.);; 2 Teng County Forestry Bureau, Guangxi Zhuang Autonomous Region, Wuzhou 543300, China

**Keywords:** *Monochamus alternatus*, restriction site-associated DNA sequencing, simple sequence repeat, genetic diversity

## Abstract

*Monochamus alternatus*, a pest posing a serious threat to coniferous species, such as *Pinus massoniana*, has had devastating effects on pine forests due to its association with *Bursaphelenchus xylophilus*. The creation of unique simple sequence repeat (SSR) primers for *M. alternatus* is crucial, as there has been little study of the species’ phylogeography. The aim of this study was to identify and create polymorphic SSR primers by sequencing samples of *M. alternatus* obtained from three different sampling points using the restriction site-associated DNA sequencing (Red-seq) approach. Subsequently, supplementary samples were integrated, and genetic typing was performed utilizing the identified polymorphic primers. Through comprehensive analysis, a total of 95,612 SSR loci were identified. Among these, mononucleotide repeats (51.43%), dinucleotide repeats (28.79%), and trinucleotide repeats (16.74%) predominated among the SSR motif types. Ultimately, 18 pairs of SSR primers were screened out, demonstrating stable amplification and high polymorphism. Genetic typing revealed that the mean number of alleles (Na) for these primer pairs ranged from 3 to 8, observed heterozygosity (Ho) ranged from 0.133 to 0.733, polymorphic information content (PIC) ranged from 0.294 and 0.783, and Shannon’s index (I) ranged from 0.590 to 1.802. This study effectively produced 16 pairs of SSR primers that can be applied to different populations of *M. alternatus*. As a result, important tools for furthering studies on the phylogeography of pine wood nematodes, creating genetic maps, gene mapping, and carrying out in-depth investigations into gene function have been made available.

## Simple Summary

Pine woods have suffered major damage from the beetle *Monochamus alternatus*, which spreads the nematode *Bursaphelenchus xylophilus* and poses a serious threat to conifers like Pinus massoniana. In order to fill the knowledge gap regarding the phylogeography of *M. alternatus*, specific simple sequence repeat (SSR) primers were designed as part of this work. To find polymorphic SSRs, researchers sequenced *M. alternatus* specimens from three different locations using the Red-seq approach. After additional analysis, 95,612 SSR loci were found, most of which were repeats of mononucleotide (51.43%), dinucleotide (28.79%), and trinucleotide (16.74%) repeats. Eighteen pairs of SSR primers were selected for their stability and high polymorphism, with genetic typing showing an average allele count of 3–8, heterozygosity (Ho) from 0.13 to 0.73, PIC values ranging from 0.29 to 0.78, and Shannon’s index between 0.59 and 1.80. The study resulted in 16 usable SSR primer pairs, enhancing research capabilities in phylogeography, genetic mapping, and functional genomics of *M. alternatus*.

## 1. Introduction

The *Monochamus alternatus*, an incredibly destructive wood-boring pest, has inflicted significant harm upon pine forest ecosystems, posing a grave threat to the survival of coniferous species such as *Pinus massoniana*, *P. koraiensis*, and *P. elliottii* [1,2]. Its larvae burrow deep into trees, interrupting growth and inflicting lasting harm to wood quality. [3]. Compounding the problem is its function as the major vector of *Bursaphelenchus xylophilus*, which accelerates pine tree mortality and causes significant forest resource losses [4,5]. With the growing consequences of global warming and the rapid extension of artificial forest regions, *M. alternatus*’s scope of damage has continued to rise unchecked. Consequently, urgent measures are imperative to bolster prevention and control efforts against *M. alternatus*, safeguarding the sustainable utilization of forest resources and ensuring the health and stability of the ecological environment.

Molecular markers, vital tools in genetic and molecular biology research, play pivotal roles in species identification, genetic mapping, gene localization, and genetic diversity analysis owing to their efficient and precise characteristics [6,7]. Among these markers, simple sequence repeat (SSR) molecular markers have garnered favor among researchers in recent years due to their high polymorphism, co-dominant inheritance, and ease of detection [8]. SSR markers are widely dispersed throughout genomes, and slip-strand mutations during replication lead to significant polymorphism among individuals or populations [9]. Currently, SSR molecular markers find extensive applications across various research domains, including insect species identification, genetic diversity assessment, population genetic structure analysis, and gene localization. Leveraging SSR molecular markers, researchers can swiftly and accurately discern genetic variations among individuals or populations, thereby elucidating genetic diversity, population differentiation, and evolutionary processes within species [10,11,12]. This furnishes robust technical support and data for the in-depth exploration of scientific inquiries such as biodiversity, ecological adaptation, and species evolution.

As the breadth and severity of pine wilt disease continue to increase, posing significant threats to worldwide pine forest resources, natural landscapes, and ecological environments, the search for an efficient, environmentally friendly, and highly specialized control strategy becomes increasingly imperative [13,14]. In this dire scenario, *M. alternatus* emerges as a key focus in control endeavors as the primary vector of *B. xylophilus*. The development of SSR customized to *M. alternatus* holds enormous promise for studying the population genetic pathways of this beetle. It permits accurate identification and monitoring of *M. alternatus* populations, allowing for a better understanding of their distribution patterns, migration routes, and reproductive behaviors. These insights are critical for developing scientifically effective control measures. As a result, investigating SSR specific to *M. alternatus* is critical for mitigating the severity of existing pine wilt illness and protecting the Earth’s pine forest resources and ecological balance. Hence, this study will leverage restriction site-associated DNA sequencing (RAD-seq), an ideal technology for SSR information research, to conduct a comprehensive analysis of SSR information in *M. alternatus* and develop polymorphic molecular markers based on this data.

## 2. Materials and Methods

### 2.1. Collection of Samples

**Figure 1:**
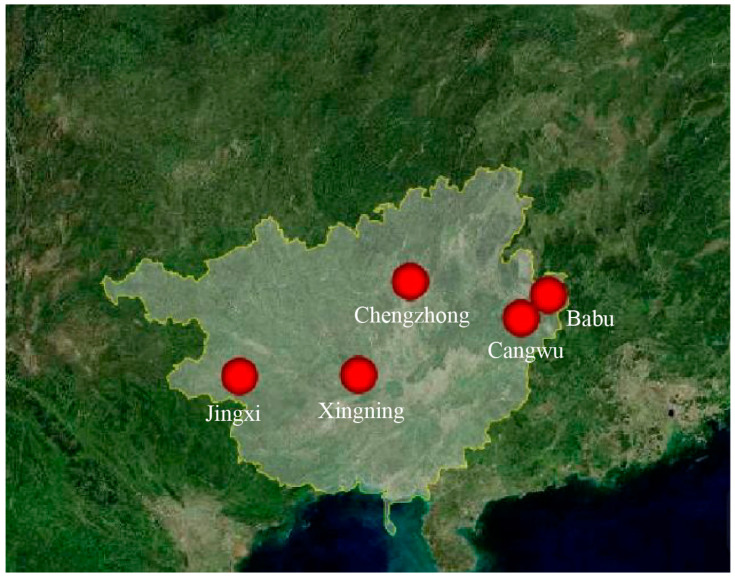
Sample information table of *M. alternatus*. Note: The location in the picture is within Guangxi Zhuang Autonomous Region, People’s Republic of China (the yellow line). The 5 red circles in the figure represent the 5 collection sites.

The sampling location is in Guangxi Zhuang Autonomous Region, People’s Republic of China. During the period spanning from July to September 2023, we implemented *M. alternatus* traps within select pine forest regions in the Guangxi Zhuang Autonomous Region, China (Figure 1). Live *M. alternatus* specimens captured were meticulously transferred into 2.0 mL sterile centrifuge tubes and preserved by adding anhydrous ethanol. These samples were subsequently stored in a −80 °C freezer at the School of Forestry, Guangxi University (Guangxi, China), to support future research endeavors.

### 2.2. Genomic DNA Extraction and Library Construction

For the extraction of genomic DNA from the thoracic muscles of individual *M. alternatus* specimens, we employed the EasyPure^®^ Genomic DNA Kit (Beijing Transgen Biotech Co., Ltd., Beijing, China) according to the instructions. Subsequently, we meticulously assessed the concentration and quality of the extracted DNA using 1% agarose gel electrophoresis and a NanoDrop ultraviolet spectrophotometer(Thermo Fisher Scientific, Waltham, MA, USA). Only DNA samples meeting stringent concentration and quality criteria were chosen and preserved in a −20 °C freezer for subsequent applications. Moreover, we selected *M. alternatus* samples from JX, XN, and CW, which are geographically distanced and endemic areas for pine wilt disease, to extract genomic DNA. These selected samples were then entrusted to Wuhan Tianyi Huayu Gene Technology Co., Ltd. for library construction and subsequent RAD sequencing.

### 2.3. High-Quality Data Acquisition and Sequence Integration

To ensure the precision and credibility of the data, we utilized Fast QC (version 0.11.7, http://www.bioinformatics.babraham.ac.uk/projects/fastqc, accessed on 3 December 2023) for comprehensive quality control. Subsequently, we employed Fastp (v0.20.0) software to meticulously filter the raw data using a sliding window approach and remove the linker sequence, low-quality read segments, sequences with high uncertainty in base information rate, and short sequences to obtain high-quality, effective data. Following this, we integrated the data obtained from the three samples for further processing. During the sequence assembly phase, we employed FLASH software (version 1.2.11, Magoc and Salzberg 2011) to merge the sequences into single high-quality reads based on the principle of overlap. In this process, we specified the following parameters: a minimum overlap length (Min overlap) of 10, a maximum mismatch density (Max mismatch density) of 0.20, and banned the formation of “outie” pairings (Allow “outie” pairs: false).

### 2.4. SSR Search and Result Compilation

To precisely pinpoint SSR loci within all sequences, we used the SSR Identification Tool (MISA) (http://pgrc.ipk-gatersleben.de/misa/ accessed on 6 December 2023) to search for SSR loci in the RAD-seq dataset. During the search process, we configured the following parameters: mono-10, di-6, tri-5, tetra-5, penta-5, and hexa-5. Furthermore, to thoroughly identify SSRs within complex sequences, we allowed a maximum distance of 100 bp between two distinct SSRs. Additionally, we categorized reverse complementary and shifted permutation sequences as the same class of SSRs to ensure accuracy and comprehensiveness in identification.

### 2.5. SSR Primer Design

To design primers targeting SSR sequences within clusters with a polymorphism greater than 2, we employed primer3 v2.3.6 software. During the design process, particular attention was paid to the length and position of the target fragments, ensuring that the amplified target fragment length was controlled between 100 and 400 bp, with the position extending from the first base before the repeat sequence to the fifth base after the repeat sequence. Other settings were set to their default values. Several filtering stages were carried out after the primer design. First, SSR types with compound repeat motifs and single nucleotide repeat motifs were not included. Second, in order to exclude potential interference from a second SSR during the amplification process, only SSR types containing a single SSR on each read were chosen for primer construction in order to assure the correctness of the polymorphism. Furthermore, primer design was limited to particular clusters and needed polymorphism of ≥2 for SSRs inside these clusters. Each designed primer had to be backed by at least two primers inside the clusters in order to assure primer reliability and prevent sequencing mistakes. Lastly, all primer results that were identical were eliminated.

### 2.6. Genotyping and Genetic Diversity Analysis

Fluorescent dyes 5′-FAM, 5′-HEX, and 5′-ROX were added to the developed primers at the 5′ end of the upstream primer. Following that, 15 samples from four populations—XN, CW, CZ, JX, and BB—were subjected to PCR amplification. After polymorphic primers were found, fifteen more samples were added for PCR amplification. The PCR reaction mixture (total volume 25 μL) comprised the following: 1 μL of forward primer (F), 1 μL of reverse primer (R), 1 μL of genomic DNA, and 22 μL of 1.1× T3 Super PCR Mix (Beijing Qingke Biological Technology Co., Ltd, Beijing, China). The PCR program was configured as follows: initial denaturation at 98 °C for 3 min, followed by cycling with denaturation at 98 °C for 15 s, annealing at 53~55 °C for 15 s, extension at 72 °C for 15 s, and a final extension at 72 °C for 2 min. PCR products underwent analysis via 1% agarose gel electrophoresis, and upon verification of distinct bands, they were forwarded to Wuhan Tianyi Huayu Genetic Technology Co., Ltd. for capillary electrophoresis-based genotyping. Subsequently, the genotyping data were systematically organized in Excel sheets based on loci order. Utilizing Popgene 1.32 software (https://sites.ualberta.ca/~fyeh/popgene_download.html, accessed on 12 December 2023), allele number (Na), effective allele number (Ne), Shannon’s index (I), observed heterozygosity (Ho), and expected heterozygosity (He) were computed for each locus. Additionally, the polymorphic information content (PIC) for each locus was calculated using SSR Toolkit v3.1.1 software.

## 3. Results

### 3.1. Sequencing Quality Assessment

Following RAD-seq and rigorous filtering processes, we collected sequencing data from three samples, each providing 6.32 to 6.87 million high-quality sequences (Table 1). These sequences displayed an average base content ranging from 900 million to 980 million, indicating a considerable sequencing depth. The quality assessment found an extraordinarily high average proportion of high-quality sequences, 96.33%, while the average proportion of high-quality bases was 95.28%, confirming the overall quality of the sequencing data. Further analysis of sequencing quality indicators revealed an average Q20 value of 97.4% and an even higher average Q30 value of 97.71%, demonstrating the exceptionally high sequencing accuracy attained. Furthermore, the average GC content was 38.95%, which falls within the usual range and increases the credibility of the sequencing results. Finally, by combining these sequencing results, we created a dataset including 2.58 million read pairs, accounting for 25.90% of all read pairs acquired.

### 3.2. Distribution Characteristics of SSR Loci

Out of a total of 2,577,819 read pairs, we successfully identified 95,612 SSR loci. Among these, 83,097 sequences contained SSR loci, with 18,986 sequences exhibiting flanking sequence lengths exceeding 20 bp. Furthermore, 12,115 sequences with multiple SSR loci were found, bringing the total number of compound SSR loci to 12,325. The most common SSR motif types were found to be dinucleotide repeats, trinucleotide repeats, and mononucleotide repeats; these motif types accounted for 51.43%, 28.79%, and 16.74% of the SSR loci that were found. In contrast, tetranucleotide repeats, pentanucleotide repeats, and hexanucleotide repeats were relatively rare, with frequencies of 1.50%, 0.45%, and 1.06%, respectively. Further scrutiny of the more frequent SSR motifs unveiled 2 types of mononucleotide repeats, 4 types of dinucleotide repeats, and 10 types of trinucleotide repeats. Among the mononucleotide repeats, the A/T motif predominated, accounting for 98.9%, while the C/G motif was less common, representing only 1.1%. For dinucleotide repeats, the AT/AT motif emerged as the most prevalent, occupying 87.37% of the total, followed by the AG/CT motif and AC/GT motif at 6.09% and 5.72%, respectively, all surpassing the proportion of the CG/CG motif. Among the trinucleotide repeats, the AAT/AAT motif exhibited the highest frequency at 77.34%, while the frequencies of the other nine motifs were all below 5% (Table 2).

### 3.3. Screening of SSR Primers

For the purpose of amplifying the complete genome of *M. alternatus* by PCR, 192 pairs of SSR primers were chosen at random from four distinct sampling points. Of the primer pairs, 94 were able to accomplish complete amplification during the amplification process, while 98 primer pairs showed partial amplification failure. To ensure study accuracy and efficacy, sequences with low allele numbers (Na ≤ 1) and poor peak quality (too much meaningless peak) were removed. After this thorough filtering method, we finally acquired 16 pairs of polymorphic primers (Table 3). Among the 16 pairs of polymorphic primers obtained, SSR motif types with various lengths showed distinct distributions. Specifically, there was only one SSR location containing pentanucleotide repetitions, nine SSR loci containing trinucleotide repeats, and six SSR loci containing dinucleotide repeats.

### 3.4. Analysis of Polymorphic of SSR Loci

**Figure 2:**
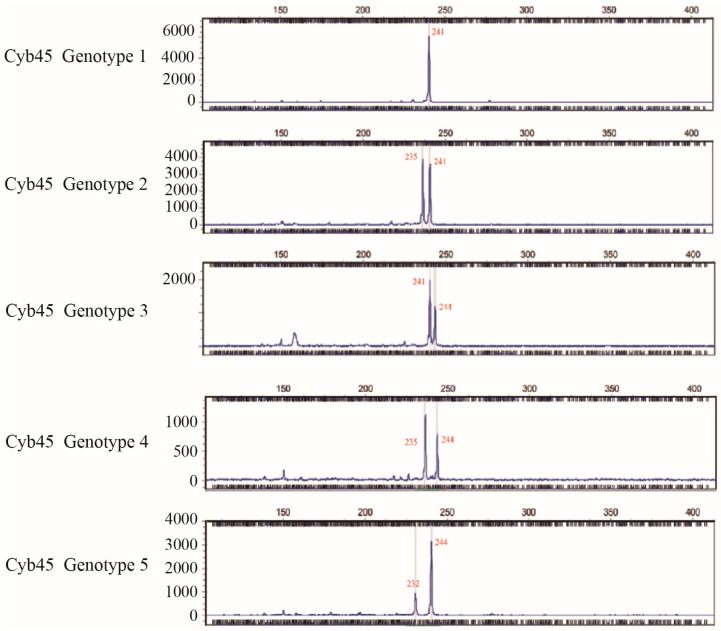
Capillary electrophoresis profile of five genotypes of primer Cyb45. The abscissa represents the allele, and the ordinate represents the peak height.

*M. alternatus* samples from five different sampling points were amplified using the carefully selected 16 SSR primers, and the genotyping outcomes were satisfactory. Among them, Cyb45 had four alleles and five distinct genotypes (Figure 2). The genotyping results vividly illustrate the diverse characteristics of each genetic locus. Specifically, the number of alleles (Na) at each locus ranged from 3 to 8, while the effective number of alleles (Ne) spanned from 1.50 to 5.23. Among the loci, Cby047 displayed the lowest effective number of alleles, whereas Cby070 showcased the highest effective number of alleles. Further exploration of the genetic diversity indices of these loci unveiled a range of Shannon’s index (I) values from 0.59 to 1.80, with a mean of 1.17, signifying varying degrees of genetic information content at these loci. The observed heterozygosity (Ho) fluctuated from 0.13 to 0.73, with a mean of 0.41, portraying the distribution of heterozygosity within the samples. Expected heterozygosity (He) ranged between 0.33 and 0.81, with a mean of 0.60, providing crucial insights for evaluating population genetic structure. Additionally, the gene diversity (Nei’s) spanned from 0.67 to 0.90, with a mean of 0.80, underscoring the high genetic diversity of the *M. alternatus* population. The polymorphic information content (PIC), a pivotal indicator of locus informativeness, varied from 0.29 to 0.78, with a mean of 0.55, highlighting the substantial information value of these SSR loci for genetic analysis and population studies.

## 4. Discussion

With the advent of high-throughput sequencing technologies, DNA sequencing methods related to enzyme-cutting sites have emerged as innovative tools. Among the RAD-seq, the employment of chip hybridization offers robust support for both the development and genotyping of SSRs through direct sequencing analysis [15,16]. While the methods for SSR primer development from transcriptome sequences hold a significant presence in genetic research, they entail drawbacks such as the need for substantial data volumes, labor-intensive experimental work, and relatively limited variation information [17,18]. This constraint derives from the fact that transcriptome sequences are largely found in gene coding regions, which are often highly conserved [19]. Therefore, getting adequate genetic variation information frequently involves large-scale experimental screening [18]. Conversely, RAD-seq constructed using whole-genome DNA sequences, offers broader coverage and the capacity to reveal a richer array of genetic variation information [20]. In this study, we successfully obtained RAD-seq of *M. alternatus* from three different sampling locations utilizing RAD-seq technology. The high-quality sequences obtained boasted an average Q20 value of 97.4% and a Q30 value of 97.71%, confirming the correctness and dependability of the sequencing results. Leveraging this dataset, we conducted an exhaustive analysis of SSR locus information in *M. alternatus*. Out of a total of 2,577,819 read pairs, we identified 95,612 SSR loci. SSR polymorphism is substantially influenced by their length, with longer SSRs exhibiting more polymorphisms (≥ 20 bp). It is noteworthy that 22.84% of SSRs in *M. alternatus* exceed 20 bp in length, indicating the potential for high genetic diversity within the species. This high genetic diversity may confer strong invasive capabilities to *M. alternatus*, making it easier for them to establish in invaded areas and perhaps inflicting ecological damage.

The majority of the unearthed SSR loci in this study (51.43%) were found to have mononucleotide repeat motifs, which were closely followed by dinucleotide repeat motifs (28.79%) and trinucleotide repeat motifs (16.74%). Notably, the frequencies of A/T, AT/AT, and AAT/ATT motifs were particularly pronounced, indicating a distinct AT bias in the base composition of *M. alternatus*. Several studies on SSRs in insects have revealed a wide variety of SSR locus types, with notable differences in the primary repeat types amongst species. Examples of such species include *Phenacoccus solenopsis* and *Anticarsia gemmatalis*, which are primarily known for their mononucleotide repeat motifs; *Ochthebius lejolisii* and *O. quadricollis*, which are primarily known for their dinucleotide repeat motifs; *Anopheles darlingi* and *Tomicus yunnanensis*, which are primarily known for their trinucleotide repeat motifs; *Anoplophora glabripennis*, which is primarily known for its occurrences; and so on [21,22,23,24,25,26]. We speculate that this differential phenomenon may be attributed to the genetic characteristics inherent to each species. Furthermore, differences in SSR retrieval parameters, choice of transcriptome sequencing platforms, and variations in data processing software could also influence the observed results [27]. In this study, we meticulously screened 192 pairs of primers with good polymorphic specificity, among which 16 pairs efficiently amplified clear, stable, and polymorphic-rich specific bands. This demonstrates the practical applicability of SSR primers created for *M. alternatus*, particularly across various populations of the species.

This study conducted genetic diversity analysis on *M. alternatus* from four distinct sampling sites. The analysis of the data reported in Table 4 demonstrated that *M. alternatus* specimens from the four sampling sites had higher genetic diversity. Further analysis revealed that the polymorphic information content (PIC) ranged from 0.29 to 0.78, with the majority of loci having PIC values exceeding 0.50, indicating highly polymorphic loci [28]. This finding revealed that the 16 primer pairs used for this investigation were effective at gathering genetic variation information. As a result, an appropriate subset of these primers might be chosen to act as useful genetic markers for measuring genetic diversity among *M. alternatus* populations from various geographic locations. A comparison with the genetic diversity analysis results of *M. alternatus* from various places in Japan by Kagaya et al., based on SSR loci, indicated consistency in the number of alleles at SSR loci but higher observed heterozygosity [29]. This finding further affirms the stability and polymorphic advantages of the primers used in our study. Moreover, studies by Hu et al. and Fu et al. also indicated substantial genetic diversity in *M. alternatus* across different ranges [30,31]. Previous research has also demonstrated significant genetic diversity of *M. alternatus* within provinces due to human activities. In the future, building upon the SSR loci obtained in this study, we aim to further investigate the genetic diversity of *M. alternatus* within provinces to elucidate population genetic structure and genetic differentiation characteristics. This endeavor seeks to provide a deeper and more comprehensive understanding of the ecology and genetics of *M. alternatus*.

## 5. Conclusions

This study utilized a restriction-site associated DNA sequencing technique to develop 16 pairs of SSR primers for *M. alternatus*, which were applied to most populations of this species. The amplification performance of the 16 primer pairs on the *M. alternatus* was excellent, achieving 100% amplification efficiency across all 15 samples. Meanwhile, the rich genetic information contained within these primers renders them suitable for the analysis of genetic diversity in *M. alternatus*. This research has significant implications for advancing the construction of the genetic map, gene localization, and exploration of gene functions for *M. alternatus*.

## Figures and Tables

**Table 1:** Statistics of the filtered and RAD-seq data.

Sample	HQ Reads	HQ Reads (%)	HQ Data (bp)	HQ Data (%)	GC (%)	Q20 (%)	Q30 (%)
Xingning	6,865,024	95.97	977,699,246	94.91	44.10	97.32	92.62
Cangwu	6,722,466	96.47	957,257,257	95.40	35.11	97.38	92.62
Jingxi	6,318,574	96.56	900,244,397	95.54	37.65	97.49	92.90

Note: “HQ reads” indicates high-quality reads; “HQ reads (%)” indicates the percentage of high-quality reads in all reads; “HQ Data (bp)” represents the number of high-quality base pairs, “HQ Data (%)” represents the number of high-quality base pairs as a percentage of all base pairs; “GC” represents the ratio of G + C base pairs to all base pairs; “Q20 (%)” and “Q30 (%)” represent the percentage of bases in the original sequence with mass values greater than 20 and 30 (error rates less than 0.1%).

**Table 2:** SSR motif results and statistics of mononucleotide, dinucleotide, and trinucleotide repeat motifs.

SSR Motif Type	Repeat Type	Count	Percentage (%)	SSR Motif Number	Percentage (%)
Mononucleotide Repeat Motifs	A/T	48,535	98.9	49,179	51.43
	C/G	644	1.1		
Dinucleotide Repeat Motifs	AC/GT	1575	5.72	27,532	28.79
	AG/CT	1678	6.09		
	AT/AT	24,055	87.37		
	CG/CG	224	0.81		
Trinucleotide Repeat Motifs	AAC/GTT	404	2.52	16,012	16.74
	AAG/CTT	519	3.24		
	AAT/ATT	12,383	77.34		
	ACC/GGT	198	1.24		
	ACG/CGT	214	1.34		
	ACT/AGT	606	3.78		
	AGC/CTG	420	2.62		
	AGG/CCT	306	1.91		
	ATC/ATG	620	3.87		
	CCG/CGG	342	2.14		
Tetranucleotide Repeat Motifs	AAAT/ATTT	572	39.86	1435	1.50
	ACAG/CTGC	390	27.18		
Pentanucleotide Repeat Motifs	AAAAT/ATTTT	154	35.24	437	0.45
Hexanucleotide Repeat Motifs	AATGAC/ATTGTC	237	23.30	1017	1.06
	AAGATG/ATCTTC	124	12.19		
	AATGAT/ATCATT	113	11.11		

**Table 3:** The 16 pairs of polymorphic SSR primer sequences for the *M. alternatus*.

Primer	F-Primer-R-Primer	Repeat Primitives and Repeat Times	Destination Fragment Size
Cby018	5′-TTCGACAAGTAGAGGAAAAGGAA-3′ 5′-GCGGACAGGCTAACTTTGAG-3′	(TAT)23	199
Cby020	5′-TCAGCAAAATGGAAATCACG-3′ 5′-AAGTGGCACCTTTGAAACAA-3′	(TG)8	183
Cby021	5′-TTTGCAACAAAATTCTCCACA-3′ 5′-CACCCTATAGCTCTGTGAGCG-3′	(AT)10	162
Cby028	5′-CTGAGTGTGAACTTCCCCTTG-3′ 5′-GTTGTGAGGGCAACGAACTT-3′	(TTA)7	171
Cby039	5′-ACATTGCAAGTGGTGGGTCT-3′ 5′-TATGTTGGTGCTGCCACTGT-3′	(AT)6	165
Cby045	5′-CAACCTGATGCCAAGTTGAA-3′ 5′-GGGACCACGTTACTTGCATT-3′	(AAT)6	219
Cby047	5′-TGGCACATCATGCTTCTTTT-3′ 5′-TTCTGGACAAATCCTCGTGA-3′	(TAA)5	120
Cby054	5′-AGCAAAGCCCCAACTGTTTA-3′ 5′-TTTGTTTCGTTTTCTAGGTTGACA-3′	(TG)6	215
Cby055	5′-AATATGCAGGAAAAGTGCGG-3′ 5′-GCTCGTAATTAGCATGAGGGG-3′	(ATA)7	187
Cby061	5′-GTCGTTGTTGCGGTTCTTTT-3′ 5′-AACGTTCTCAACGGCCAC-3′	(TCG)7	152
Cby063	5′-CACTGAATGGTGGTTAGGGC-3′ 5′-CCAAAGAAGATGTAGAACCATCC-3′	(TAT)6	194
Cby066	5′-TCCAAACGGAGAAATTGTGA-3′ 5′-GACCGTCACCATAAGCCTGT-3′	(AT)6	121
Cby069	5′-ACACCGTCATAAAAGGCGAG-3′ 5′-CTTCTGGGCTAATCCCTGTG-3′	(AAT)6	187
Cby070	5′-ATTCAGCTGCCTCCATGAAA-3′ 5′-CGGCCAAAATAAAGCAGGAT-3′	(TAAAA)7	212
Cby086	5′-ATTCCGTTTTTCCCTGGACT-3′ 5′-ACTTGGTGGCGACCTTAATA-3′	(TAA)5	160
Cby089	5′-GGATGGAAAAACACATCAGTCA-3′ 5′-CTGTTTGGGACACGCCTTAT-3′	(TG)6	179

**Table 4:** SSR primer polymorphism information.

Locus	Na	Ne	Si	Ho	He	Nei’s	Pic
Cby018	4	2.43	1.01	0.15	0.59	0.79	0.50
Cby020	7	4.94	1.76	0.27	0.80	0.90	0.77
Cby021	8	4.83	1.80	0.73	0.79	0.90	0.77
Cby028	4	1.93	0.85	0.53	0.48	0.74	0.42
Cby039	3	2.42	0.97	0.27	0.59	0.79	0.51
Cby045	4	1.65	0.80	0.30	0.39	0.70	0.37
Cby047	3	1.49	0.59	0.40	0.33	0.67	0.29
Cby054	5	2.59	1.15	0.67	0.61	0.81	0.55
Cby055	7	2.53	1.32	0.47	0.61	0.80	0.58
Cby061	4	3.17	1.24	0.23	0.69	0.84	0.63
Cby063	3	1.59	0.64	0.40	0.37	0.69	0.32
Cby066	4	2.41	1.10	0.47	0.58	0.79	0.54
Cby069	4	2.43	1.01	0.40	0.59	0.79	0.50
Cby070	7	5.23	1.77	0.13	0.81	0.90	0.78
Cby086	7	3.76	1.59	0.53	0.73	0.87	0.70
Cby089	4	2.22	1.04	0.53	0.55	0.78	0.51

Note: Na, Number of allele; Ne, effective number of allele; Si, Shannon Index; Ho, heterozygosity of observation; heterozygosity of expected; Nei’s, Nei’s gene diversity; Pic, polymorphism information content.

## Data Availability

The datasets generated and/or analyzed during this study are available from the corresponding author on reasonable request.
